# Hydrate of Chloral as a Remedial Agent—Effects of Its Continued Use

**Published:** 1871-06

**Authors:** 


### ARTICLE VI.—*Hydrate of Chloral as a Remedial Agent—Effects of its Continued Use.* By * * C.

It is with a very genuine hesitation and reluctance that I venture to offer to the readers of the *Journal* a few facts, illustrating the effects of the continued use of the above-named remedy, as observed in one special case, though not thereby inferring that they would be similar in every other case. I am not only a non-professional writer, but also a Southerner, and consequently, if the sage exposition (more learned than wise) of Dr. McElroy* is to be believed, my "molecular forms of structure" vary so much from the normal Yankee standard, and from those of my possible readers, as perhaps to differentiate wholly our method of inductive reasoning. But by sticking to simple facts, the language of which is pretty much the same everywhere, I may render myself intelligible. This remedy (hydrate of chloral) being now in a manner on its trial before the profession, any instances of unmistakable facts, illustrating peculiar effects, may not be useless.

The patient, whose case I propose to refer to, is a lady who abandoned quite lately the use of opium, after indulging moderately in what is termed the "opium habit" for several years. Suffering in consequence from obstinate wakefulness and distressing nervous irritability, she was advised, by a physician of the highest professional standing, to use hydrate of chloral, as potass. bromide, which she had previously taken, did not seem to meet the indications. She commenced using the chloral on

* Vide Chicago Med. Journal, May No., p. 284.

January 1st, of this present year (entirely dropping the other remedy), beginning with thirty grains per diem, or rather per noctem, and being, at that period, in all respects, save those I have mentioned, in apparently perfect health—appetite good, digestion unimpaired, secretions and excretions all right. In about two weeks from that time—30 grs. chloral hydrate having been taken each night—the patient began to suffer from slight irritability of the stomach, and inability to retain ordinary food, which increased rapidly until the functions of this organ became so impaired that it would tolerate neither hot drinks—such as tea and coffee—nor any solid food. She suffered greatly also from burning heat and painful tenderness in the epigastric region, the least pressure causing great uneasiness. This sensation of intense heat daily increased, digestion seemed destroyed, the craving for ice-water or ice itself was continual, yet it afforded no permanent relief. In a short time after this nothing but iced sago broths or jellies could be retained by the stomach for a moment. About the twentieth of February, acute inflammation declared itself in the eyes of the patient; such was the intense heat, that tear-drops, as they formed and fell, felt to the burning and inflamed eyeballs like tiny hail-stones or drops of ice-cold water. The throbbing of the temporal artery announced determination of blood to be the immediate cause of the inflammation, as also did the bright florid color of the inflamed parts. As the inflammation increased, the secretion of tears was suspended, and there was exudation from the eyes, first of a thin transparent matter, which afterwards became translucent and acquired greater consistency. It must have been highly fibrinous, for when partially dried on the eye-lashes, it could easily be drawn out, and by a slight twisting motion of the fingers, actually spun into short threads. Comparatively little abnormal matter was thrown up from the stomach, yet the appearance of that which was thus expelled, was really remarkable. It resembled from first to last very minute pieces of yellowish white and very thin blotting paper, somewhat curled or crimped, floating in a thin translucent fluid. One of these small floating particles I placed under a magnifying power of 10,000 diameters, and saw innumerable fibrillæ crossing each other at every conceivable angle, and apparently adhering together, though not very tenaciously. Symptomatic fever did not precede, but followed the inflammation. It was characterized by a pulse feeble yet hard, often jerking, skin dry and hot, with occasional sweats at irregular intervals; secretions suppressed; weakness of patient extreme at times, yet alternated with fits of great feverish excitement. The poor patient had unluckily read some of the so-called professional writings of Dr. Ludlow, till on this one subject (the opium habits, and its effects on tissue and the vital organs) her mind was fairly befogged. For a long time she actually supposed these phenomena to be a necessary sequelæ to the abandonment of opium, until observing the steady onward march of the inflammation, with its train of attendant evils, foreboding danger and death, her brain rallied sufficiently from its fatalistic nonsense to secrete a modicum of common sense, which immediately suggested that this sort ot thing had gone far enough—that other than merely palliative treatment was demanded, and that as a first step it might be well to discontinue the use of such a powerful stimulant as chloral hydrate. Common sense was listened to, and, to the lady's surprise, in less than forty-eight hours after the chloral was discontinued, the inflamed organs experienced sensible relief. I have not alluded to the excessive *pain* which the patient felt in the eyes and the stomach, for I supposed every reader would infer the presence of pain from the fact of intense inflammation. This pain abated; and, skipping over successive steps, I may say that from that day the process of recovery commenced, some simple hygienic remedies being used to facilitate its progress. The organs of secretion resumed their functions, fever diminished and finally disappeared, and in little over two weeks' time, the inflammation and every trace of it had vanished, the *vis medicatrix naturæ* having, almost entirely unassisted, accomplished the cure in this pure and healthy organization as soon as the irritant cause was removed. Poultices of slippery elm powder and rosewater applied to the eyes, diluent drinks, and occasionally a mild aperient taken inwardly, with frequent bathing to equalize the circulation, comprised the whole treatment. If I except a remaining inability to secure regular and natural sleep, the patient is now in excellent health.

Have we not good reason to suppose that the chloral hydrate was the exciting cause of all this inflammation, acting, no doubt, as a chemical irritant?

Whether it acted locally, that is, directly upon the affected organs, as a disciple of "solidism" would perhaps assert; whether it entered into the circulation as a *materies morbi*, carrying with it irritation, and perhaps altering by degrees the tonicity of the arteries; or whether, lastly, its primary effect was on the nerves, communicating by sympathy with the vascular system, I must leave to better pathologists than myself to determine. Yet, it does not seem very unreasonable to infer, from the knowledge we have as to its resultant effects upon the blood, that continued use of an agent like chloral might, in time, produce destructive metamorphosis of the delicate nerve tissue, fed and sustained, as it would be, by this chemically altered blood. But it hardly becomes me to offer any crude hypothesis of my own, addressing, as I am, those who are so greatly my superiors in scientific knowledge.

I would merely add—

1st. In no previous illness of this patient had there ever been any appearance of inflammatory diathesis.

2d. From first to last no other remedy (except the simples I have mentioned) was used but chloral hydrate.

3d. The patient whose case I have reported was the writer of this article, who is therefore sure of her *facts*, even if wrong in her inferences.

It is, lastly, only justice to say that the physician who prescribed the chloral did *not* do so as a "specific," but merely to meet the indications of the case at the time; nor is he responsible for the continued use of it, since residing in a distant city, he was not again consulted by his former patient.

